# Emotional, hyperactivity and inattention problems in adolescents with immunocompromising chronic diseases during the COVID-19 pandemic

**DOI:** 10.1016/j.clinsp.2023.100167

**Published:** 2023-01-24

**Authors:** Reinan T. Campos, Livia Lindoso, Renan A. de Sousa, Alberto C. Helito, Bianca P. Ihara, Claudia A.A. Strabelli, Levi M.V. Paradelas, Beatriz O.L. Carneiro, Maria Paula R. Cardoso, Jean Paulo V. de Souza, Marianna R. de M. Freire, Camilla Astley, Moisés F. Laurentino, Izabel M. Buscatti, Katia Kozu, Nadia E. Aikawa, Adriana M.E. Sallum, Juliana C.O. Ferreira, Juliana R. Simon, Vivianne S.L. Viana, Ligia B. Queiroz, Bruno Gualano, Hamilton Roschel, Rosa Maria R. Pereira, Ricardo K. Toma, Andréia Watanabe, Patricia M. Grangeiro, Caio B. Casella, Sylvia C. Farhat, Guilherme V. Polanczyk, Lucia Maria A. Campos, Clovis A. Silva

**Affiliations:** Hospital das Clínicas da Faculdade de Medicina, Universidade de São Paulo (HCFMUSP), São Paulo, SP, Brazil

**Keywords:** COVID-19, Mental health, Adolescent, Chronic disease

## Abstract

**Objective:**

To assess factors associated with emotional changes and Hyperactivity/Inattention (HI) motivated by COVID-19 quarantine in adolescents with immunocompromising diseases.

**Methods:**

A cross-sectional study included 343 adolescents with immunocompromising diseases and 108 healthy adolescents. Online questionnaires were answered including socio-demographic data and self-rated healthcare routine during COVID-19 quarantine and validated surveys: Strengths and Difficulties Questionnaire (SDQ), Pittsburgh Sleep Quality Index (PSQI), Pediatric Quality of Life Inventory 4.0 (PedsQL4.0).

**Results:**

The frequencies of abnormal emotional SDQ scores from adolescents with chronic diseases were similar to those of healthy subjects (110/343 [32%] vs. 38/108 [35%], p = 0.548), as well as abnormal hyperactivity/inattention SDQ scores (79/343 [23%] vs. 29/108 [27%], p = 0.417). Logistic regression analysis of independent variables associated with abnormal emotional scores from adolescents with chronic diseases showed: female sex (Odds Ratio [OR = 3.76]; 95% Confidence Interval (95% CI) 2.00‒7.05; p < 0.001), poor sleep quality (OR = 2.05; 95% CI 1.08‒3.88; p = 0.028) and intrafamilial violence during pandemic (OR = 2.17; 95% CI 1.12‒4.19; p = 0.021) as independently associated with abnormal emotional scores, whereas total PedsQL score was inversely associated with abnormal emotional scores (OR = 0.95; 95% CI 0.93‒0.96; p < 0.0001). Logistic regression analysis associated with abnormal HI scores from patients evidenced that total PedsQL score (OR = 0.97; 95% CI 0.95‒0.99; p = 0.010], changes in medical appointments during the pandemic (OR = 0.39; 95% CI 0.19-0.79; p = 0.021), and reliable COVID-19 information (OR = 0.35; 95% CI 0.16‒0.77; p = 0.026) remained inversely associated with abnormal HI scores.

**Conclusion:**

The present study showed emotional and HI disturbances in adolescents with chronic immunosuppressive diseases during the COVID-19 pandemic. It reinforces the need to promptly implement a longitudinal program to protect the mental health of adolescents with and without chronic illnesses during future pandemics.

## Highlights

• The frequencies of abnormal emotional SDQ scores from adolescents with chronic diseases were significantly lower than those of healthy controls during the COVID-19 pandemic.
• The frequencies of abnormal hyperactivity and inattention SDQ scores from adolescents with chronic diseases were significantly lower than those of healthy controls.
• Abnormal emotional scores from adolescents with chronic diseases were as independently associated with female sex, poor sleep quality, and intrafamilial violence during the pandemic.
• Abnormal hyperactivity and inattention SDQ scores from patients were inversely associated with total PedsQL score, changes in medical appointments during the pandemic, and reliable COVID-19 information remained.

## Introduction

The novel Coronavirus Disease 2019 (COVID-19) has had numerous consequences.^1^ Despite a low death incidence among adolescents,^2^^,^^3^ home confinement, school restrictions and social isolation due to quarantine/lockdown brought a great impact on this population.^4^, ^5^, ^6^, ^7^

Indeed, socializing with peers represents a fundamental stimulus for cognitive development, identity formation, and mental health promotion in adolescence.^8^ Studies in different parts of the world showed the negative impact on the mental health of adolescents during the COVID-19 pandemic.^4^ Hyperactivity was another physiological issue during this period, particularly increased by confinement.^9^^,^^10^

Additional concerns affected adolescent populations with complex and systemic chronic diseases during this infectious disease.^11^, ^12^, ^13^ These findings are more relevant in those with immunosuppressive chronic diseases, due to possible disease complications, flares, severe infections, multiple hospitalizations, low economic status, physical and mental impact, as well as a decrease in Health-Related Quality of Life (HRQL) parameters.^8^^,^^14^

Emotional and hyperactivity were relevant issues during the COVID-19 pandemic.^4^^,^^9^^,^^10^ Recently, the authors compared physical and mental health impacts during COVID-19 quarantine in two groups of adolescents: with chronic illnesses and healthy adolescents, and only the total score of Strengths and Difficulties Questionnaire (SDQ) was evaluated to assess mental health impact.^15^^,^^16^ This instrument is a mental health screening questionnaire, with specific domains, and the authors observed that approximately one-third of both groups had a negative impact on the total SDQ score.^15^ However, the authors did not evaluate for this population^15^^,^^16^ the specific emotional and hyperactivity/inattention domains on SDQ scores, as well as a concomitant analysis of HRQL and sleep quality parameters from a vulnerable population and healthy controls.

Thus, the aims of this study were to analyze the factors associated with emotional changes and hyperactivity/inattention aspects during the quarantine in adolescents with immunosuppressing chronic diseases and healthy controls. The authors also evaluated demographic data, changes in routine due to the pandemic, HRQL, and sleep quality parameters from both groups.

## Materials and methods

From July to October 2020, the authors carried out a cross-sectional study with adolescents between 10 and 18 years old. During this period isolation and quarantine measures were adopted due to the COVID-19 pandemic. The authors invited 555 adolescents with immunocompromising chronic diseases, residents in São Paulo, followed up in a tertiary and university hospital in Brazil, and 149 healthy adolescents. Two hundred and twelve patients were excluded due to: refusal to participate in the study (n = 114) or failure to answer the survey properly (n = 98), and n = 343 patients remained in the study group. Healthy teenagers were invited (n = 149) through media and social media. After parental approval and exclusion of unanswered (n = 23) or incomplete forms (n = 18), 108 healthy adolescents remained as the control group. Before participation in this study, consent and assent terms were signed online by legal guardians and adolescents respectively. This study was permitted by the National Research Ethics Committee (CONEP n° 4.081.961).

Adolescents were diagnosed with the following chronic immunocompromising diseases, according to specific criteria: autoimmune rheumatic diseases (juvenile idiopathic arthritis, juvenile systemic lupus erythematosus, and juvenile dermatomyositis), kidney diseases (glomerulopathies, stages 4 and 5 chronic kidney disease and kidney transplantation), gastrointestinal and liver diseases (eosinophilic esophagitis, inflammatory bowel disease, celiac disease, autoimmune hepatitis disease, and liver transplantation).^15^^,^^16^

Data collection was done through electronic forms. These were accessible through cell phones, tablets or computers and had a mean response time of 45 minutes. To improve the survey response rate, the questionnaire was sent to each at least 6 times by email or Whatsapp® for each participant. The Research Electronic Data Capture (REDCaps®) was used to collect and organize the study data, hosted at the University of Sao Paulo.

The study included four steps including answers about the last month before study entry. The first step was a semi-structured questionnaire including questions about socioeconomic and educational data, care routines, sleep, physical activity, pandemic information, and COVID-19 quarantine impacts, as previously described.^15^^,^^12^ The semi-structured questionnaire is available as an appendix.^16^ Furthermore, three validated instruments were used to evaluate mental health, sleep quality, and HRQL.

Mental health was assessed by a Brazilian-Portuguese validated version of the Strengths and Difficulties Questionnaire (SDQ).^18^ This self-report tool is composed of 25 items separated into five scales: emotional symptoms, conduct problems, hyperactivity/inattention, peer relationship problems, and prosocial behavior. Answers were categorized in “not true”, “somewhat true” and “certainly true”. Each item generated a score ranging from 0 to 2. Two groups were divided according to the more recent four-band of self-reported SDQ: abnormal (cut-off points “high/low” and “very high/very low”) and normal/borderline (cut-off points “close to average” and “slightly raised/slighted lowered”).^19^^,^^20^ The present study included a specific analysis of two SDQ scales: emotional SDQ and Hyperactivity/Inattention (HI) SDQ scores. For emotional SDQ scores, abnormal values ranged from 6 to 10. The hyperactivity/inattention scores were categorized as abnormal with values between 7 to 10.^19^^,^^20^

The Brazilian-Portuguese version of the Pittsburg Sleep Quality Index (PSQI-PT) was used to assess sleep quality, including 19 items to assess different aspects of sleep quality. Each item can be scored from 0 to 3, with a maximum total score of 21. Poor sleep quality is defined by scores greater than 5.^21^

HRQL was measured using the Pediatric Quality of Life Inventory 4.0 (PedsQL), which is an instrument composed of 23 items that encompass physical, social, emotional, and school aspects. The score ranges from 0 to 100, being that higher scores are associated with better HRQL.^22^

### Statistical analysis

The sample size of 425 adolescents provided a power of 80% to find differences greater than 10.4% of abnormal emotional and abnormal HI SDQ scores among adolescents with chronic immunosuppressing diseases and healthy controls (GraphPad StatMate 1.01, GraphPad Software, Inc., CA, USA). Statistical analyses were carried out with Statistical Package for Social Sciences (SPSS) for Windows 24.0 (IBM Corp., Armonk, NY, USA). The results were presented as numbers (frequencies) for categorical variables and as median (minimum value and maximum value) or mean ± standard deviations for continuous variables. The scores that had a normal distribution were compared using Student's *t*-test and those with non-normal distribution were compared using the Mann-Whitney test. Differences in categorical variables were evaluated using Fisher's exact test or Pearson's Chi-Square test, as indicated. Logistic regression analysis models were done using abnormal emotional or abnormal hyperactivity/inattention SDQ scores as a dependent variable and variables that presented a statistical significance level of p < 0.2 in the univariate analyses as independent variables, in adolescents with chronic immunocompromising diseases and healthy controls. Statistical significance was set at p < 0.05.

## Results

Diagnoses of associated mental diseases in adolescents with chronic diseases before the COVID-19 pandemic occurred in 31/343 (9%). None of the healthy adolescents reported previous diagnoses of mental disorders. Table 1 includes SDQ scores reported by adolescents with chronic diseases versus healthy adolescents during quarantine of the COVID-19 pandemic. The frequencies of abnormal emotional SDQ scores from adolescents with chronic diseases were similar to those of healthy subjects (110/343 [32%] vs. 38/108 [35%], p = 0.548), as well as abnormal hyperactivity/inattention SDQ scores (79/343 [23%] vs. 29/108 [27%], p = 0.417) (Table 1).

**Table 1:** Strengths and Difficulties Questionnaire (SDQ) scores reported by adolescents with chronic diseases versus healthy adolescents during quarantine of the coronavirus infectious disease 2019 (COVID-19) pandemic.

SDQ domains (score)	Adolescents with chronic diseases (n = 343)	Healthy adolescentes (n = 108)	p
Current age	14 (10‒18)	15 (10‒18)	0.733
Female sex	67 (60)	44 (40)	0.970
Total difficulties score (0‒40)	14 (0‒32)	14 (1‒36)	0.519
Abnormal total difficulties score	103 (30)	34 (32)	0.775
Peer problems (0‒10)	2 (0‒10)	2 (0‒10)	0.359
Emotional problems (0‒10)	4 (0‒10)	5 (0‒10)	0.164
Emotional disorders	110 (32)	38 (35)	0.548
Conduct problems (0‒10)	2 (0‒9)	2 (0‒8)	0.321
Hyperactivity/inattention (0‒10)	5 (0‒10)	5 (0‒9)	0.046
Hyperactivity/inattention disorder	79 (23)	29 (27)	0.417
Prosocial (0‒10)	8 (0‒10)	8 (1‒10)	0.957
Impact score (0‒10)	0 (0‒10)	1 (0‒8)	0.052

Results are presented in n (%), and median (minimum-maximum values).

### Abnormal emotional SDQ, PSQI-PT test and PedsQL scores

Further analysis comparing adolescents with chronic diseases and abnormal emotional scores to healthy adolescents with abnormal emotional scores during the COVID-19 pandemic showed that the median age (15 [10‒18] vs. 15 [10‒18] years, p = 0.964) and female sex (81% vs. 76%, p = 0.544) were similar in both groups. The frequency of public-school students (81% vs. 60%, p = 0.008) and adolescents who take care of elderly people (p = 0.017) was significantly higher in the former group, contrasting with lower frequencies of school homework during the COVID-19 pandemic (p = 0.004) and sleep duration < 8 hs/day (46% vs. 66%, p = 0.039).

Comparison between PSQI-PT and PedsQL scores reported by adolescents with chronic diseases and abnormal emotional scores compared to healthy adolescents with abnormal emotional scores during the COVID-19 pandemic showed that school performance (55 [0‒100] vs. 65 [30‒100], p = 0.004) was significantly reduced among adolescents with chronic diseases.

Table 2 shows the comparison between patients with chronic diseases with abnormal emotional scores versus normal/borderline emotional scores of the SDQ score. The former group had significantly higher frequencies of the female sex (81% vs. 52%, p < 0.001), more than 3 household members (75% vs. 65%, p = 0.048), and public-school students (81% vs. 71%, p = 0.029). The frequencies of increase in screen time (92% vs. 84%, p = 0.041) and exposure to intrafamily violence (32% vs. 15%, p < 0.001) were also significantly higher among adolescents with abnormal emotional scores, as well as the median of fear of COVID in VAS (7 [0‒10] vs. 6 [0‒10], p = 0.019) and fear of underlying disease activity/complication in VAS (8 [0‒10] vs. 5 [0‒10], p < 0.001).

**Table 2:** Demographic data, information, and impact of the Coronavirus Infectious Disease 2019 (COVID-19) pandemic reported by adolescents with chronic diseases and an abnormal emotional score versus adolescents with chronic diseases and normal/borderline emotional score during quarantine.

Variables	Adolescents with chronic diseases and abnormal emotional score (n = 110)	Adolescents with chronic diseases and normal/borderline emotional score (n = 233)	p
**Socio-demographic**			
Current age	15 (10‒18)	14 (10‒18)	0.082
Female sex	89 (81)	122 (52)	**<0.001**
Caucasians	57 (52)	119 (51)	0.897
> 5 rooms in the residence	46 (42)	102 (44)	0.732
> 3 household's members in the residence	83 (75)	151 (65)	**0.048**
**Educational data**			
Attending school before COVID-19 pandemic	95 (86)	119 (85)	0.813
Level of schooling			0.132
Elementary school	58 (52)	114 (62)	
Middle school	46 (42)	68 (29)	
High school	4 (4)	14 (6)	
Not studying	2 (2)	7 (3)	
Public school adolescents	90 (81)	165 (71)	**0.029**
School homework during COVID-19 pandemic			0.071
No homework	25 (23)	32 (14)	
≤ 3 hours/day	46 (42)	96 (41)	
> 3 hours/day	39 (35)	105 (45)	
**Healthcare routine during the pandemic**			
Medical appointment during the pandemic			0.343
Discontinued	37 (36)	76 (34)	
Decreased	44 (43)	83 (38)	
Unchanged	21 (21)	62 (28)	
Forgetting to take your medication			**0.016**
Without forgetting	58 (59)	152 (74)	
1‒2 days	32 (33)	48 (23)	
3 days	8 (8)	6 (3)	
**General information on the COVID-19 pandemic**			
COVID-19 information source			0.181
Family and friends	11 (10)	15 (6)	
Health professionals	8 (7)	9 (4)	
Social media/television/radio	91 (83)	209 (90)	
Reliable COVID-19 information	75 (68)	179 (77)	0.232
Compliance to the “stay-home” policy	104 (95)	226 (97)	0.363
**VAS (0**‒**10)**			
Fear of COVID	7 (0‒10)	6 (0‒10)	**0.019**
Fear of underlying disease activity/complication	8 (0‒10)	5 (0‒10)	**<0.001**
**Impact of COVID-19 quarantine**			
Household members with COVID-19	23 (21)	25 (11)	**0.011**
Life routine changed after the “physical distancing” policy	104 (95)	213 (91)	0.307
Housework			**0.001**
No housework	20 (18)	62 (27)	
≤ 1 hours/day	42 (38)	116 (50)	
> 1 hours/day	48 (44)	55 (23)	
Adolescents who take care of elderly people			0.094
Not taking care	86 (78)	173 (74)	
≤ 1 hours/day	6 (6)	30 (13)	
> 1 hours/day	18 (16)	30 (13)	
Physical activity per week	1.3 (0‒10)	2.7 (0‒10)	**0.007**
Sleep duration < 8 h/day	51 (46)	68 (29)	**0.002**
Sleep after midnight	76 (69)	137 (59)	0.067
Sleep quality	6.7 (0‒10)	8.8 (0‒10)	**<0.001**
Screen time			0.056
<3 hours/day	11 (10)	24 (10)	
4‒6 hours/day	40 (36)	115 (49)	
≥7 hours/day	59 (54)	94 (41)	
Screen time increased during pandemic	101 (92)	195 (84)	**0.041**
Alcohol use during pandemic			0.381
Increased	1 (1)	0 (0)	
Did not change	1 (1)	2 (1)	
Decreased	2 (2)	2 (1)	
Do not drink alcohol	106 (96)	299 (98)	
Financial status during the pandemic			0.096
Worsen	48 (44)	75 (32)	
Did not change	58 (53)	151 (65)	
Improve	4 (3)	7 (3)	
Household members working outside of home	100 (91)	191 (83)	0.059
Intrafamilial violence during pandemic	35 (32)	34 (15)	**<0.001**

Results are presented in n (%), median (minimum‒maximum values), VAS, Visual Analogue Scale in the last month (scale 0–10).

Adolescents with chronic diseases and abnormal emotional scores had poor sleep quality on PSQI-PT score more often than those with normal/borderline emotional scores (56% vs. 30%, p < 0.001). The median PSQI-PT total score values and the vast majority of its domains were significantly higher in the first group (p < 0.05). The median of the total scale PedsQL score was significantly reduced among adolescents with chronic diseases and abnormal emotional scores (64 [21‒89] vs. 78 [29‒100], p < 0.001), likewise physical domain (68 [13‒100] vs. 81 [6‒100], p < 0.001) and psychosocial health summary scores (58 [13‒97] vs. 75 [20‒100], p < 0.001) (Table 3).

**Table 3:** Pittsburgh Sleep Quality Index (PSQI) and Pediatric Quality of Life Inventory 4.0 (PedsQL) sores reported by adolescents with chronic diseases and abnormal emotional score versus adolescents with chronic diseases and normal/borderline emotional score during quarantine of Coronavirus Infectious Disease 2019 (COVID-19) pandemic.

Domains	Adolescents with chronic diseases and abnormal emotional score (n = 110)	Adolescents with chronic diseases and normal/borderline emotional score (n = 233)	p
PSQI score			
PSQI total score (0‒21)	6.57±3.66	4.59±3.13	<0.001
Poor sleep quality, total score > 5	53 (56)	64 (30)	<0.001
Sleep latency (0‒3)	1.41±1.09	0.99±0.97	0.001
Sleep duration (0‒3)	0.25±0.69	0.16±0.57	0.267
Sleep efficiency (0‒3)	0.75±1.12	0.43±0.84	0.008
Sleep disturbances (0‒3)	1.28±0.54	0.98±0.46	<0.001
Sleep medication use (0‒3)	0.70±1.21	0.69±1.18	0.928
Overall sleep quality (0‒3)	1.16±0.88	0.75±0.72	<0.001
Day dysfunction due to sleepiness (0‒3)	1.02±0.84	0.59±0.73	<0.001
PedsQL score			
Total scale score (0‒100)	64 (21‒89)	78 (29‒100)	<0.001
Physical health summary score (0‒100)	68 (13‒100)	81 (6‒100)	<0.001
Psychosocial health summary score (0‒100)	58 (13‒97)	75 (20‒100)	<0.001
Emotional functioning (0‒100)	40 (0‒100)	70 (15‒100)	<0.001
Social functioning (0‒100)	80 (10‒100)	90 (20‒100)	<0.001
School functioning (0‒100)	55 (0‒100)	70 (0‒100)	<0.001

Results are presented as medians (minimum‒maximum values), means±standard deviations, and n (%).

Table 4 shows the logistic regression analysis of independent variables associated with abnormal emotional scores among adolescents with chronic diseases. Female sex (Odds Ratio [OR] = 3.76; 95% Confidence Interval [95% CI] 2.00‒7.05; p < 0.001), poor sleep quality (OR = 2.05; 95% CI 1.08‒3.88; p = 0.028) and intrafamilial violence during the pandemic (OR = 2.17; 95% CI 1.12‒4.19; p = 0.021) remained independently associated with abnormal emotional scores, whereas total PedsQL score was inversely associated with abnormal emotional scores (OR = 0.95; 95% CI 0.93‒0.96; p < 0.0001). The R² of the Nagelkerke test was 0.383.

**Table 4:** Final models of logistic regression analyses to evaluate independent variables associated with abnormal emotional score in adolescents with chronic diseases during quarantine.

Independent variables	Odds ratio	95% CI	P
Female sex	3.76	2.00‒7.05	**<0.001**
Public School adolescents	0.59	0.31‒1.15	0.121
Household members with COVID-19	2.12	0.99‒4.51	0.053
Physical activity per week	1.00	0.99‒1.01	0.523
Intrafamilial violence during pandemic	2.17	1.12‒4.19	**0.021**
Total PedsQL score	0.95	0.93‒0.96	**<0.001**
Sleep duration < 8 h/day	1.15	0.61‒2.17	0.666
Sleep quality	0.99	0.98 ‒1.01	0.409
Poor sleep quality	2.05	1.08‒3.88	**0.028**

CI, Confidence Interval, R² of the Nagelkerke test = 0.383.

### Abnormal hyperactivity/inattention SDQ, PSQI-PT test and PedsQL scores

Comparison between adolescents with chronic diseases and abnormal hyperactivity/inattention scores versus healthy adolescents with abnormal hyperactivity/inattention scores during the quarantine showed that school homework was significantly reduced in the first group (p = 0.045). No differences were evidenced in PSQI-PT and PedsQL scores between the two groups (p > 0.05).

The median fear of underlying disease activity/complication by VAS was significantly higher in adolescents with abnormal hyperactivity/inattention scores versus those with normal/borderline hyperactivity/inattention (7 [0‒10] vs. 5 [0‒10], p = 0.005). The frequencies of changes in medical appointments during the pandemic (p = 0.014) and intrafamilial violence were also significantly higher in the former group (34% vs. 16%, p < 0.001). On the other hand, the frequency of reliable COVID-19 information was significantly lower in the first group (61% vs. 78%, p = 0.003) (Table 5).

**Table 5:** Demographic data, information, and impact of Coronavirus Infectious Disease 2019 (COVID-19) pandemic reported by adolescents with chronic diseases and abnormal Hyperactivity/Inattention (HI) score versus adolescents with chronic diseases and normal/borderline HI score during quarantine.

Variables	Adolescents with chronic diseases and abnormal HI score (n = 79)	Adolescents with chronic diseases and normal/borderline HI score (n = 264)	p
**Socio-demographic**			
Current age	14 (10‒18)	15 (10‒18)	0.294
Female sex	55 (70)	156 (59)	0.092
Caucasians	42 (53)	134 (51)	0.707
> 5 rooms in the residence	34 (43)	114 (43)	0.982
> 3 household's members in the residence	51 (65)	183 (69)	0.425
**Educational data**			
Attending school before COVID-19 pandemic	71 (90)	223 (85)	0.229
Level of schooling			0.197
Elementary school	52 (66)	150 (57)	
Middle school	22 (28)	92 (35)	
High school	5 (6)	13 (5)	
Not studying	0 (0)	9 (3)	
Public school adolescents	54 (68)	201 (76)	0.165
School homework during COVID-19 pandemic			0.676
No homework	11 (14)	46 (17)	
≤ 3 hours/day	32 (41)	110 (42)	
> 3 hours/day	36 (45)	108 (41)	
**Healthcare routine during the pandemic**			
Medical appointment during the pandemic			**0.014**
Discontinued	36 (49)	77 (31)	
Decreased	23 (32)	104 (42)	
Unchanged	14 (19)	69 (27)	
Forgetting to take your medication			0.060
Without forgetting	39 (59)	171 (72)	
1‒2 days	25 (38)	55 (23)	
3 days	2 (3)	12 (5)	
**General information of COVID-19 pandemic**			
COVID-19 information source			0.243
Family and friends	6 (8)	20 (8)	
Health professionals	1 (1)	16 (6)	
Social media/television/radio	72 (91)	228 (86)	
Reliable COVID-19 information	48 (61)	206 (78)	**0.003**
Compliance to “stay-home” policy	75 (95)	226 (97)	0.363
**VAS (0-10)**			
Fear of COVID	7 (0‒10)	6 (0‒10)	0.339
Fear of underlying disease activity/complication	7 (0‒10)	5 (0‒10)	**0.005**
**Impact of COVID-19 quarantine**			
Household members with COVID-19	12 (15)	36 (14)	0.727
Life routine changed after the “physical distancing” policy	74 (94)	243 (92)	0.634
Housework			0.188
No housework	24 (30)	58 (22)	
≤ 1 hours/day	30 (38)	128 (48)	
> 1 hours/day	25 (32)	78 (30)	
Adolescents who take care of elderly people			0.530
Not taking care	60 (76)	199 (76)	
≤ 1 hours/day	6 (8)	30 (11)	
> 1 hours/day	13 (16)	35 (13)	
Physical activity per week	1.1 (0‒10)	2.8 (0‒10)	**0.008**
Sleep duration < 8 h/day	40 (51)	79 (30)	**0.001**
Sleep after midnight	56 (71)	157 (60)	0.067
Sleep quality	6.3 (0‒10)	8.7 (0‒10)	**<0.001**
Screen time			0.600
< 3 hours/day	8 (10)	27 (10)	
4‒6 hours/day	32 (41)	123 (47)	
≥ 7 hours/day	39 (49)	114 (43)	
Screen time increased during pandemic	71 (90)	225 (85)	0.292
Alcohol use during pandemic			0.316
Increased	0 (0)	1 (0)	
Did not change	2 (3)	1 (0)	
Decreased	1 (1)	3 (2)	
Do not drink alcohol	76 (96)	259 (98)	
Financial status during the pandemic			**0.007**
Worsen	40 (51)	83 (31)	
Did not change	37 (47)	172 (65)	
Improve	2 (2)	9 (4)	
Household members working outside of home	72 (91)	222 (84)	0.116
Intrafamilial violence during pandemic	27 (34)	42 (16)	**<0.001**

Results are presented in n (%), median (minimum‒maximum values), VAS, Visual Analogue Scale in the last month (scale 0–10).

The median of PSQI-PT total score was significantly higher among chronic disease adolescents with abnormal hyperactivity/inattention scores versus those with normal/borderline hyperactivity/inattention (6.01 ± 3.35 vs. 4.94 ± 3.40, p = 0.019). Furthermore, the same group had higher means of sleep disturbance (1.23 ± 0.51 vs. 1.03 ± 0.49, p = 0.003), overall sleep quality (1.15 ± 0.81 vs. 0.78 ± 0.77, p = 0.001) and day dysfunction due to sleepiness (0.97 ± 0.85 vs. 0.65 ± 0.75, p = 0.004) (Table 6). The median of all scores of PedsQL was significantly reduced in adolescents with abnormal hyperactivity/inattention scores compared to those with normal/borderline hyperactivity/inattention scores (p < 0.05) (Table 6).

**Table 6:** Pittsburgh Sleep Quality Index (PSQI) and Pediatric Quality of Life Inventory 4.0 (PedsQL) sores reported by adolescents with chronic diseases and abnormal Hyperactivity/Inattention (HI) score versus adolescents with chronic diseases and normal/borderline HI score during quarantine of Coronavirus Infectious Disease 2019 (COVID-19) pandemic.

Domains	Adolescents with chronic diseases and abnormal HI score (n = 79)	Adolescents with chronic diseases and normal/borderline HI score (n = 264)	p
**PSQI score**			
PSQI total score (0‒21)	6.01±3.35	4.94±3.40	**0.019**
Poor sleep quality, total score > 5	35 (47)	82 (35)	0.065
Sleep latency (0‒3)	1.13±0.95	1.11±1‒05	0.860
Sleep duration (0‒3)	0.19±0.59	0.18±0.62	0.962
Sleep efficiency (0‒3)	0.56±0.95	0.51±0.95	0.693
Sleep disturbances (0‒3)	1.23±0.51	1.03±0.49	**0.003**
Sleep medication use (0‒3)	0.77±1.27	0.67±1.17	0.540
Overall sleep quality (0‒3)	1.15±0.81	0.78±0.77	**0.001**
Day dysfunction due to sleepiness (0‒3)	0.97±0.85	0.65±0.75	**0.004**
**PedsQL score**			
Total scale score (0‒100)	64 (28‒94)	75 (21‒100)	**<0.001**
Physical health summary score (0‒100)	69 (13‒100)	81 (6‒100)	**<0.001**
Psychosocial health summary (0‒100)	62 (23‒88)	73 (13‒100)	**<0.001**
Emotional functioning (0‒100)	45 (5‒100)	65 (0‒100)	**<0.001**
Social functioning (0‒100)	80 (25‒100)	90 (10‒100)	**<0.001**
School functioning (0‒100)	50 (0‒85)	70 (0‒100)	**<0.001**

Results are presented as medians (minimum‒maximum values), means±standard deviations, and n (%).

Table 7 shows the logistic regression analysis of independent variables associated with abnormal hyperactivity/inattention scores in adolescents with chronic diseases. Total PedsQL scores (OR = 0.97; 95% CI 0.95‒0.99; p = 0.010), changes in medical appointments during the pandemic (OR = 0.39; 95% CI 0.19‒0.79; p = 0.021), and reliable COVID-19 information (OR = 0.35; 95% CI 0.16‒0.77; p = 0.026) remained inversely associated with abnormal HI scores. The R² of the Nagelkerke test was 0.260.

**Table 7:** Final models of logistic regression analyses to evaluate independent variables associated with adolescents with chronic diseases and abnormal hyperactivity/inattention score during quarantine.

Independent variables	Odds ratio	95% CI	P
Physical activity per week	0.99	0.99‒1.01	0.657
Total PedsQL score	0.97	0.95‒0.99	**0.010**
Changes in medical appointment during the pandemic	0.39	0.19 ‒0.79	**0.021**
Reliable COVID-19 information	0.35	0.16‒0.77	**0.026**
Worst financial status during the pandemic	0.31	0.03‒2.97	0.097
Fear of underlying disease activity/complication	1.00	0.99‒1.02	0.299
Intrafamilial violence during pandemic	1.89	0.92‒3.87	0.084
Total PSQI score	0.92	0.82‒1.03	0.130
Sleep duration < 8 h/day	1.57	0.77‒3.20	0.217
Sleep quality	0.99	0.98 ‒1.00	0.135
Sleep problem	1.19	0.52‒2.73	0.677

CI, Confidence Interval, R² of the Nagelkerke test = 0.260.

## Discussion

The authors demonstrated abnormal emotional and abnormal HI SDQ scores in more than 20% of adolescents with chronic immunosuppressing illnesses and healthy adolescents during the COVID-19 pandemic. Female sex, poor sleep quality, and intrafamilial violence during the pandemic were associated with abnormal emotional scores, whereas HRQL was inversely associated. Additionally, HRQL, changes in medical appointments during the pandemic, and reliable COVID-19 information were inversely associated with abnormal HI scores.

Important to consider that the patient scores should be interpreted based on the comparison to controls. Surprisingly, in this study, the presence of chronic disease was not associated with worse emotional or HI outcomes. In contrast other surveys, that patients with chronic diseases were more vulnerable to mental health issues.^22^, ^23^, ^24^ One possible explanation for this divergence was the presence of medical support for these patients during the pandemic.

In addition, other reports showed unfavorable outcomes for chronic diseases in adolescents, evaluating other mental health tools.^23^ A Turkish study used the Revised Children's Anxiety and Depression Scale and demonstrated a 1.6-fold increased risk of anxiety.^27^ Another India study using the Kessler-10 Psychological Distress scale showed that the risk of psychological stress in teens with chronic diseases was 2.6 times greater compared to healthy adolescents.^28^

Pre-pandemic data showed that approximately 13.4% of children and adolescents had a mental disorder.^29^ The authors extended the previous observation and showed that adolescents with chronic immunosuppressing diseases had emotional and HI impacts during the COVID-19 quarantine.^15^ Emotional problems were present in almost one-third and HI problems in 23% of the immunosuppressed adolescents. Emotional changes using SDQ during COVID-19 were reported by approximately 11% of healthy adolescents in Indonesia and Germany.^30^^,^^31^ Regarding the HI domain of SDQ, the prevalence of HI abnormalities was 8% and 18%.^30^^,^^31^

The female sex was associated with altered emotional scores from adolescents with chronic immunosuppressive diseases. This fact was also previously described, showing a significant increase in symptoms of anxiety and depression in girls,^32^ probably due to stressful events reported by females^33^ and a greater tendency of girls to internalize problems compared to males.^33^ The authors also showed that poor sleep quality impacted emotional health in adolescents with chronic diseases. This point was shown in a Japanese study carried out with children before the COVID-19 pandemic.^34^ In this survey, emotional problems were related to sleep symptoms, longer sleep latency, and longer wakefulness after sleep onset.^34^

Importantly, another aspect observed herein was that abnormal emotional scores were seen in adolescents that reported an increase in domestic violence, as previously described.^8^ Social isolation resulting from the quarantine combined with economic impacts may induce domestic violence, as reported by another adolescent population,^35^ thus alerting to the need for a high degree of suspicion and surveillance during stressful periods.

Furthermore, abnormal HI scores occurred in adolescents with chronic diseases. A study with healthy Spanish adolescents showed higher levels of the HI score related to daily activities, such as having little schoolwork or not following daily routines.^10^ In contrast, the present study showed that HRQL, changes in medical appointments during the pandemic, and reliable COVID-19 information were inversely associated with abnormal HI scores. Indeed, HRQL parameters of teenagers were greatly impacted during the pandemic.^36^ During the COVID-19 pandemic, routine appointments, elective surgeries, and procedures were also postponed, as well as continuously hearing about information severe and lethal COVID-19 may negatively impact these adolescents.^37^

One of the principal limitations of this study was the use of a screening instrument for mental health problems, with a possible bias selection of assessing hyperactivity and emotional symptoms in the present study. The authors also did not evaluate specific instruments for depression, anxiety, and attention deficit hyperactivity disorder. The authors also did not evaluate parental physiopathology, a well-known factor that may influence emotional health in adolescents with greater previous exposure to adversities.^29^ The cross-sectional study design was also a weakness herein, since the long-term pandemic may induce fluctuations or increases in emotional problems in adolescents.^29^^,^^38^ Therefore, further prospective studies are required to evaluate the impacts of mental issues, HRQL, and sleep quality parameters.

One of the main strengths of this study was analyzing psychological changes during the COVID-19 pandemic in a diverse and vulnerable population of adolescents with chronic illnesses, living in an urban area largely affected by this infectious disease, and before COVID-19 vaccination. The authors also systematically evaluated validated instruments to assess mental issues, HRQoL, and sleep quality parameters. Another advantage of this study was the prompt access to a pediatric multidisciplinary team, especially for adolescents with physical and mental impacts, such as pediatric psychiatrists or psychologists, nurses, nutritionists, social workers, and physical educators.^16^^,^^39^^,^^40^

In conclusion, the present study showed emotional symptoms and HI among adolescents with chronic immunosuppressing diseases during the COVID-19 pandemic. Female sex, poor sleep quality, and intrafamilial violence during the pandemic were observed in those with abnormal emotional scores, whereas HRQL was inversely associated. Additionally, HRQL, changes in medical appointments during the pandemic, and reliable COVID-19 information were inversely evidenced in those with abnormal HI scores. The present study also reinforces the need to promptly implement a longitudinal program to protect the mental health of adolescents with and without chronic conditions during future pandemics.

## Authors’ contributions

All the authors contributed substantially to the conception and design of the study and to the analysis and interpretation of data. All authors revised the work critically and approved the final version.

## Funding

This study was supported by grants from Conselho Nacional de Desenvolvimento Científico e Tecnológico (CNPq 304984/2020-5 to CAS), Fundação de Amparo à Pesquisa do Estado de São Paulo (FAPESP 2015/03756-4 to CAS) and by Núcleo de Apoio à Pesquisa “Saúde da Criança e do Adolescente” da USP (NAP-CriAd) to CAS.

## Authorship criteria

All named authors approved the final draft of the manuscript, approved the submission to the Journal, and be willing to take responsibility for it in its entirety.

## Ethics committee name and study protocol number

CONEP number 4.081.961.

## Financial disclosure

The authors have no financial arrangements with a company whose product figures prominently in the submitted manuscript or with a company making a competing product.

## Conflicts of interest

The authors declare no conflicts of interest.
